# Epidemiology of *Plasmodium vivax* in Indonesia

**DOI:** 10.4269/ajtmh.16-0093

**Published:** 2016-12-28

**Authors:** Claudia Surjadjaja, Asik Surya, J. Kevin Baird

**Affiliations:** 1 ALERTAsia Foundation, Jakarta, Indonesia.; 2 Sub-Directorate for Malaria Control, Ministry of Health, Jakarta, Indonesia.; 3 Eijkman-Oxford Clinical Research Unit, Jakarta, Indonesia.; 4 Centre for Tropical Medicine, Nuffield Department of Medicine, University of Oxford, Oxford, United Kingdom.

## Abstract

Endemic malaria occurs across much of the vast Indonesian archipelago. All five species of *Plasmodium* known to naturally infect humans occur here, along with 20 species of *Anopheles* mosquitoes confirmed as carriers of malaria. Two species of plasmodia cause the overwhelming majority and virtually equal shares of malaria infections in Indonesia: *Plasmodium falciparum* and *Plasmodium vivax*. The challenge posed by *P. vivax* is especially steep in Indonesia because chloroquine-resistant strains predominate, along with Chesson-like strains that relapse quickly and multiple times at short intervals in almost all patients. Indonesia's hugely diverse human population carries many variants of glucose-6-phosphate dehydrogenase (G6PD) deficiency, most of them exhibiting severely impaired enzyme activity. Therefore, the patients most likely to benefit from primaquine therapy by preventing aggressive relapse, may also be most likely to suffer harm without G6PD deficiency screening. Indonesia faces the challenge of controlling and eventually eliminating malaria across > 13,500 islands stretching > 5,000 km and an enormous diversity of ecological, ethnographic, and socioeconomic settings, and extensive human migrations. This article describes the occurrence of *P. vivax* in Indonesia and the obstacles faced in eliminating its transmission.

## Background

Endemic transmission of the human parasite *Plasmodium vivax* occurs across most of the Indonesian archipelago.^1^ The number of clinical attacks cannot yet be estimated with precision, but probably at least several million Indonesians suffer acute vivax malaria each year. Mortality due to vivax malaria, despite the dogma of a benign character, has been documented repeatedly in Indonesian hospitals over the past decade.^2^ This parasite poses a very significant and especially difficult threat to the health of the 130 million Indonesians living at risk of this infection.^1^

The biology and ecology of *P. vivax* in Indonesia imposes relatively difficult obstacles to diagnosis, treatment, and control. There are 20 confirmed anopheline mosquito vivax malaria vector species in Indonesia scattered across a wide variety of habitats and conditions optimal for malaria transmission.^3^ Acute vivax malaria very often comes with relatively very low levels of parasitemia, thereby increasing the probability of a missed diagnosis, especially by rapid diagnostic tests.^4^ More importantly, *P. vivax* places dormant stages in the liver called hypnozoites—a single infectious anopheline bite may result in five to 15 clinical attacks over 2 years.^5^ This phenomenon amounts to malaria “transmission” without a mosquito—the silent forms in liver called hypnozoites cannot be diagnosed and do the attacking after becoming active weeks, months, or several years later. Chemotherapy of those forms, and not just those causing the acute attack, thus emerges as a crucial aspect of containing and eliminating vivax malaria.

In most of the world, chloroquine is the front-line therapy against acute vivax malaria; but in Indonesia, resistance to this drug arose, spread, and became dominant against sensitive strains.^6^ It has been abandoned in favor of artemisinin-combined therapies (ACTs). Many clinical trials evaluating those options in Indonesia found them consistently safe and effective against the acute attack by asexual blood stages of *P. vivax*. But there is, nonetheless, a serious problem with these options. Only one has been very recently confirmed as permitting safe and efficacious administration of primaquine with it (dihydroartemisinin–piperaquine [DHA-PP]).^7^ Appropriate therapy for *P. vivax* is not simply treating the acute attack, but also simultaneously preventing multiple relapses with primaquine. Demonstrating the safety and efficacy of primaquine with each new potential blood schizonticidal partner in radical cure of vivax malaria is a laborious and expensive undertaking that impedes the availability of validated therapeutic options for radical cure of *P. vivax.*

Perhaps the greatest impediment to the control and elimination of vivax malaria in Indonesia is the problem of primaquine toxicity in glucose-6-phosphate dehydrogenase–deficient (G6PDd) patients. This hugely diverse X-linked disorder is highly prevalent among many of Indonesia's hundreds of ethnic groups. The relative sensitivity to primaquine among any of these is not known, but threatening hemolytic reactions to primaquine therapy are widely known by anecdote in Indonesia.^8^ In the absence of the ability to screen patients for G6PDd where most malaria patients live, Ministry of Health policy recommends a relatively low dose of primaquine, but many providers fail to offer even that for fear of causing harm. Patients thus have limited access to primaquine and probably suffer multiple repeated attacks—each one coming with further opportunities for transmission, delayed or inappropriate therapy of the acute attack, sickness, and death. A robust point-of-care G6PDd screening device would enable a practical and effective attack upon the hypnozoite reservoir in endemic communities.

In summary, vivax malaria is a very serious and challenging health problem in Indonesia, and the National Malaria Control Program (NMCP) faces formidable technical, logistical, and financial obstacles in dealing with it. Access to safe primaquine therapy may be the most difficult and important among these. Absent such, the elimination of endemic transmission of this parasite would probably be greatly protracted and costly.

## Anopheline Vector Distribution and Ecology

**Figure 1.**
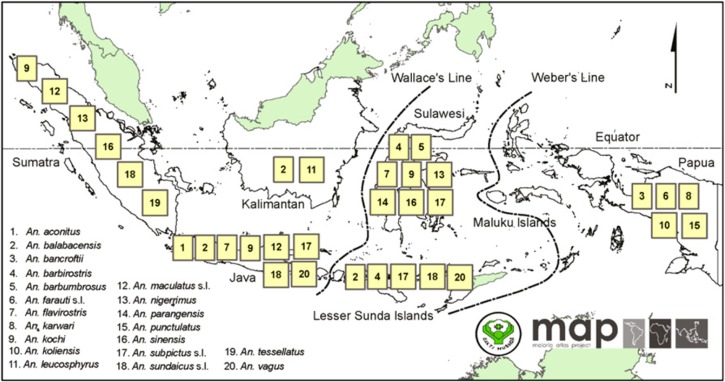
Distribution of species of anopheline mosquitoes confirmed as malaria vectors in Indonesia, reproduced from reference 3 with permission.

Two comprehensive reviews of the anophelines of Indonesia, one by Takken and others^9^ from the 1980s, and more recently by Elyazar and others,^3^ are available. In general, the human plasmodia tend to favor discreet species of anophelines, and malaria transmission tends to occur in the habitats in which those species thrive. Unlike the Greater Mekong Subregion where malaria transmission tends to be dominated by forest-dwelling anopheline vectors, the Indonesian archipelago harbors many species commonly found in plantations, aquacultural ponds, and within villages. Such species are confirmed as malaria vectors by the identification of human parasite stages within them. There is no known species of anopheline malaria vector in Indonesia that will only host *Plasmodium falciparum* but not *P. vivax*, and vice versa. If the anopheline carries one species, it is very likely to carry the other as well (data on the other more rare human plasmodia are lacking on this point). Figure 1

illustrates the complexity of anopheline distribution and ecology in Indonesia. Twenty confirmed vector species are known, each with its own distinct geographic distribution, feeding and breeding preferences, and seasonality of abundance patterns (collectively referred to as “bionomics” of the species). The reviews cited above^3^,^9^ offer encyclopedic descriptions of the bionomics of Indonesia's anopheline vectors of malaria.

## Distribution of Endemic *P. vivax*

**Figure 2.**
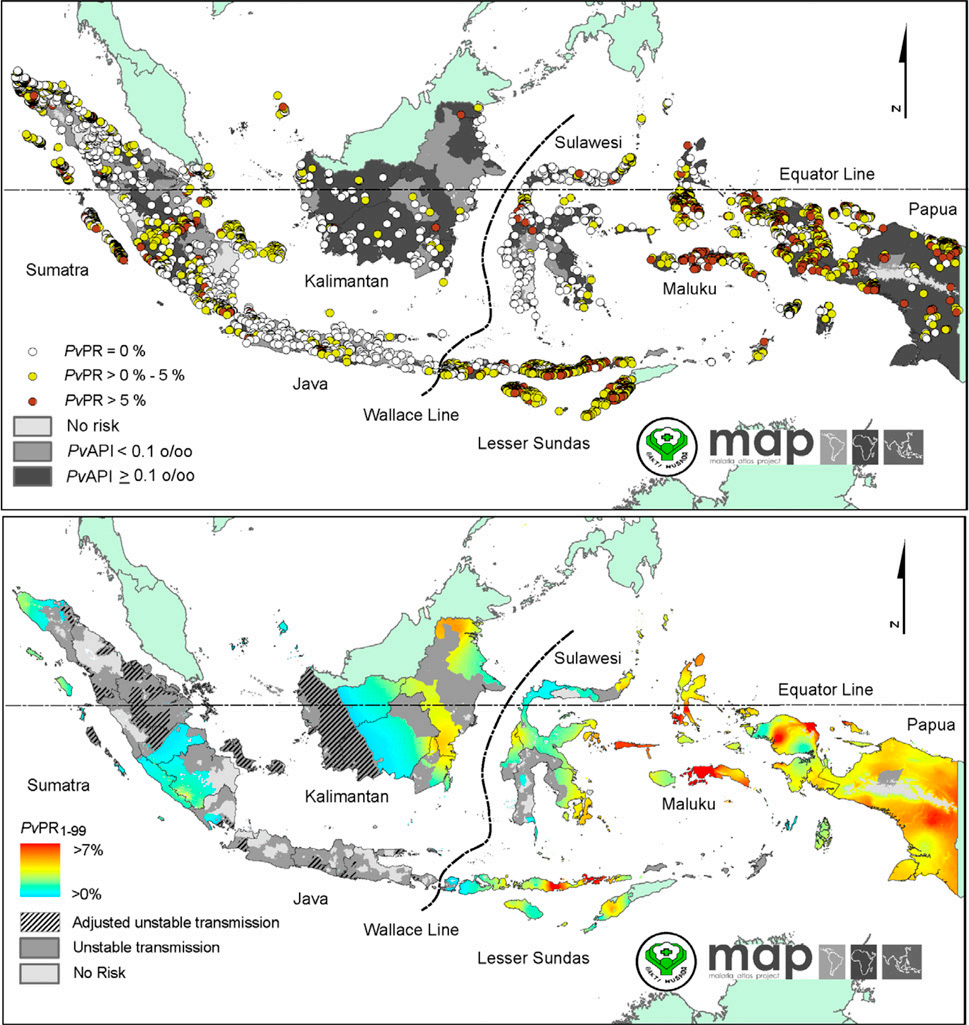
Mass blood surveys measuring parasite prevalence between 1985 and 2010 (top panel) and the modeled prevalence of *Plasmodium vivax* in Indonesia for the year 2010 (lower panel), reproduced from reference 1, published under creative commons.

Elyazar and others^1^ assembled 4,658 community-based blood surveys for malaria conducted between 1985 and 2011 to model a map of *P. vivax* prevalence (Figure 2

). In another paper, the same group^10^ summarized all known blood surveys for Indonesia (Table 1 ). In short, risk of vivax malaria occurs almost everywhere, with exceptional zones or pockets free of risk on Java, Bali, and Sumatra (and fewer pockets on Sulawesi and Papua). Most major cities in Indonesia are also free of risk, even in otherwise high-risk areas. This is because Indonesia has no urban anophelines (e.g., *Anopheles stephensi* of India). Highest risk occurs in eastern Indonesia, especially East Nusatenggara (Lesser Sundas archipelago), Maluku, and Papua. Most of Kalimantan and Sumatra also have large areas of transmission, but to a much lower level of endemicity than in most of eastern Indonesia.

The dotted line drawn through the middle of map of Figure 2, the Wallace Line, is a zoogeographic border between Asian and Australian flora and fauna, including anopheline mosquitoes (Figure 1). Although this may play some role in the contrasting malaria risks between eastern and western Indonesia, it seems more likely that the relatively poor economic development of the eastern regions drives the high risk of malaria.

## Endemic *P. vivax* Epidemiology

**Figure 3.**
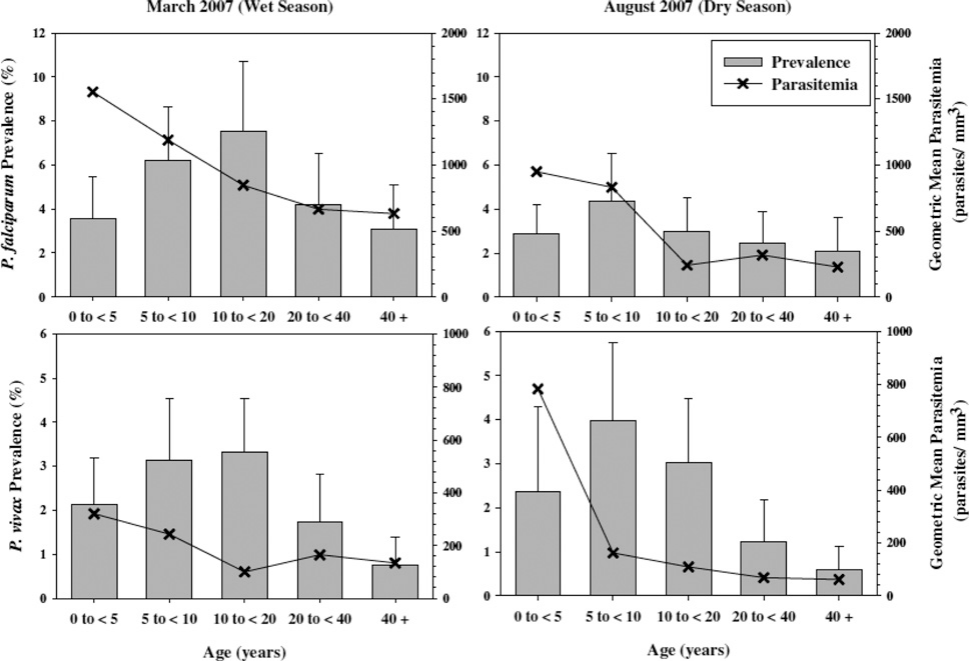
Mass blood survey findings of over 8,000 residents of western Sumba Island in eastern Indonesia during 2007, reproduced from reference 11 with permission.

The distribution of vivax malaria in the human population, where it is endemic, tends to be overwhelmingly relatively low-level transmission. Syafruddin and others^11^ conducted an exhaustive cross-sectional survey (over 8,000 slides at 45 sites) across western Sumba. Figure 3

summarizes the microscopic parasitological findings across age groups and season (wet versus dry). Western Sumba may be considered typical of the hypo- to mesoendemic malaria transmission that occurs in much of Indonesia. The prevalence of *P. vivax* (in peripheral blood smears) is uniformly low (< 5%) among age groups and varying little with seasonal rains. Sumba does have rather sharp differences in seasonal rainfall, with a distinct monsoon and dry season. The absence of sharp differences between these seasons is probably a product of hypnozoite activation (in the absence of abundant mosquitoes), as well as sufficient diversity of anopheline species to permit transmission during any season (only slight seasonal differences in prevalence for falciparum malaria). Transmission of malaria on Sumba, as in much of Indonesia, thus occurs year round with little variance according to wet versus dry season.

This prevalence survey, along with many others from all across Indonesia, indicates an important epidemiological principle with malaria—risk across demographic groups is practically invariable between the two dominant species of *Plasmodium*. This is clearly seen in Figure 3. This relative homogeneity points to equal risk of exposure by the biting anophelines carrying each species, that is, it appears to be the same mosquitoes and any resident at risk of infection by one species is essentially at equal risk of infection by the other. This has important implications regarding policy for attacking vivax malaria because a diagnosis of *P. falciparum* in such settings also carries a very high probability of carriage of *P. vivax* hypnozoites. Douglas and others^12^ in Thailand decisively demonstrated this phenomenon: 51% of patients having a primary diagnosis of *P. falciparum* experienced a relapse of *P. vivax* within just 2 months. A diagnosis of any species of plasmodia anywhere in endemic Indonesia should rationally and reasonably prompt therapy against *P. vivax* hypnozoites.^13^ Treatment policy in Indonesia does not currently recommend presumptive primaquine therapy against hypnozoites with a diagnosis of *P. falciparum* malaria, principally due to the risk of harm caused by primaquine.

**Figure 4.**
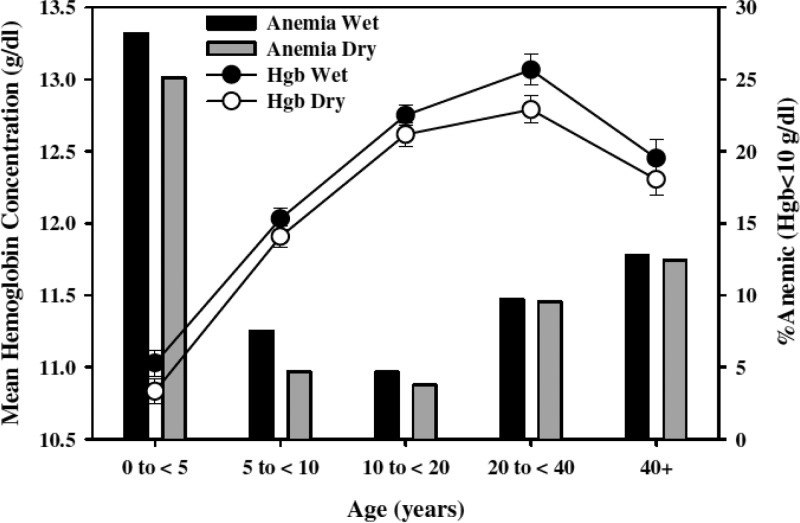
Survey of more than 8,000 residents of western Sumba in eastern Indonesia showing seasonal trends in anemia (points = mean Hb concentration; bars = percent anemia) across age groups possibly linked to malaria transmission. Reproduced from reference 11 with permission.

Prevalence on Sumba rarely exceeded 10% in even the highest risk groups. Typically, it was well below 5%. Children (but not small children) tended to have the higher prevalence, quite a bit so compared with adults, that is, 4% versus < 1%. These differences are very probably driven by naturally acquired immunity, where older residents are better able to suppress parasitemia to subpatency (no sterilizing immunity is known in malaria).^14^ Most malaria transmission in Sumba occurs within villages, especially coastal villages exposed to the efficient vector species, *Anopheles sundaicus*. The young and old are attacked with equal frequency, but the higher burden of parasites in younger age groups (both prevalence and density of parasites in blood) is reflected in the trends in hemoglobin levels reported by Syafruddin and others^11^ on Sumba (Figure 4

).

**Figure 5.**
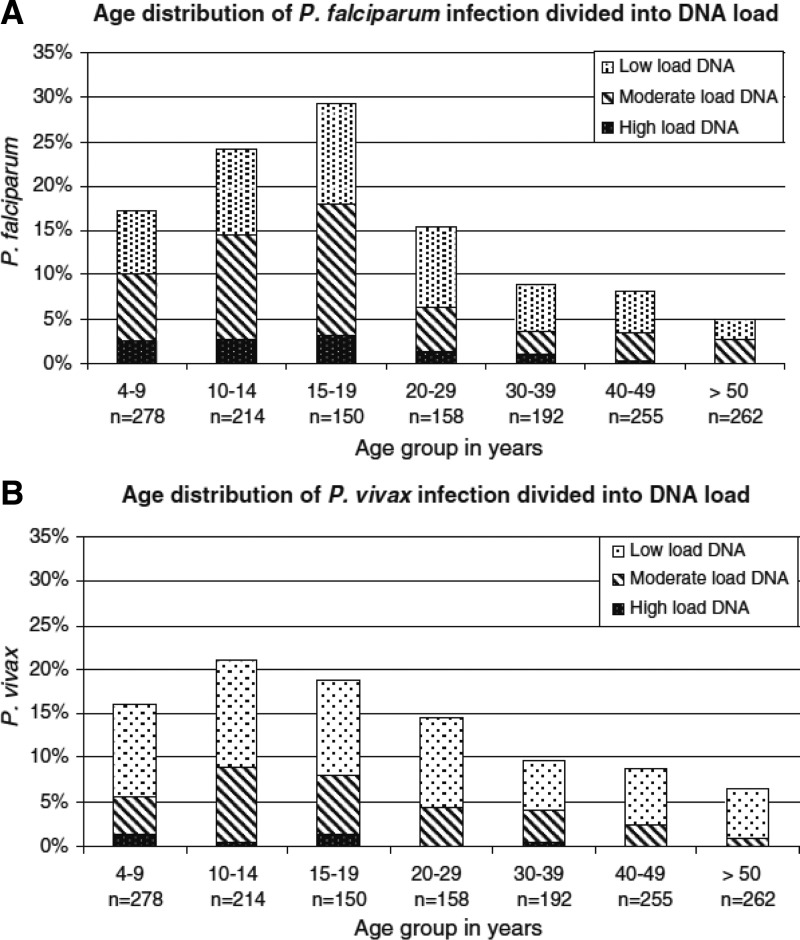
Prevalence of *Plasmodium falciparum* (top panel) and *Plasmodium vivax* (lower panel) among over 1,000 residents of Flores Island in eastern Indonesia measured by semiquantitative real time polymerase chain reaction, reproduced from reference 15 with permission.

A very important consideration in malaria epidemiology is subpatent parasitemia, that is, those beyond diagnostic reach by standard microscopy. Kaisar and others^15^ conducted a survey for malaria parasites in blood by both microscopy and real-time polymerase chain reaction (PCR) techniques at Flores Island in eastern Indonesia. In their sample of 1,509 people, 52 (3.4%) were diagnosed as having malaria by microscopic examination, whereas 399 (26%) were positive by PCR. These data, and others like it, consistently demonstrate that microscopically subpatent parasitemia is the rule in Indonesia, as elsewhere (Solomon Islands, the Mekong Region, Horn of Africa, and Amazonia).^16^ The long-held presumption that natural immunity to malaria required sustained and intense transmission needs reconsideration. This is a vitally important consideration in the context of eliminating malaria from settings of relatively low transmission intensity. Figure 5

illustrates the distribution of *P. falciparum* and *P. vivax* across age groups detected by PCR at the study site in Flores of Kaisar and others^15^

Thus, endemic malaria in Flores (and nearby Sumba and much of Indonesia) may be appreciated as largely cryptic, that is, not causing illness and staying below the level of detection by standard microscopy. Comparing the microscopic and PCR surveys exemplified here, the true prevalence of malaria, even where historically considered hypoendemic (i.e., < 5% prevalence by microscopy), may well exceed 40% in many settings. Further work and evidence on this key question is urgently needed against a backdrop of strategic planning for elimination of such possibly vast reservoirs of infection, that is, the asymptomatic and subpatent and infectious carriers of malaria who apparently dominate many hypoendemic communities.

## Outbreaks and Imported *P. vivax*

**Figure 6.**
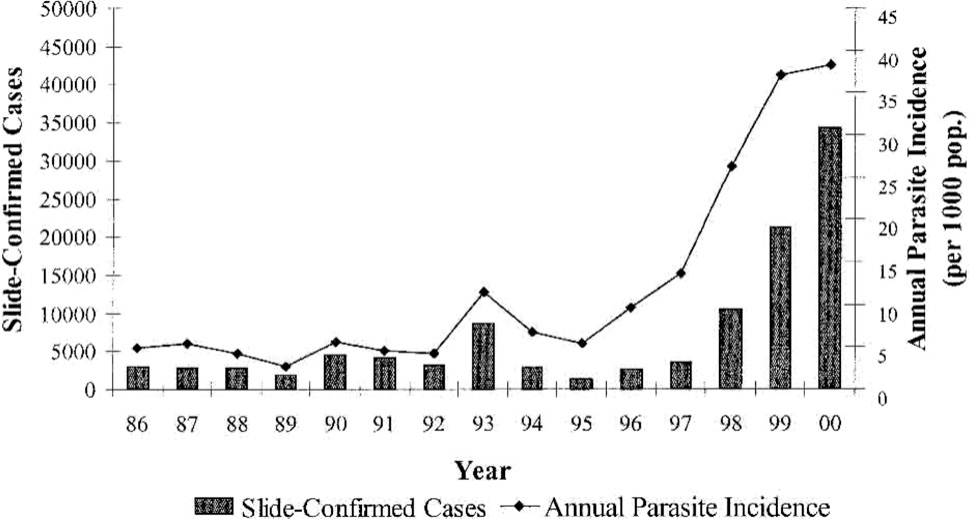
Graph illustrates progress of a malaria epidemic in the Menoreh Hills of southern Central Java including both *Plasmodium vivax* and *Plasmodium falciparum* but dominated by the latter. Reproduced from reference 18 with permission.

Excepting a few isolated pockets of stable malaria transmission, the island of Java is largely free of malaria transmission.^17^ Nonetheless, much of the island to the south of its volcanic spine remains highly receptive to malaria. These very few remaining foci of transmission sometimes cause wider epidemics. An example of this occurred in the Menoreh Hills just to the west of Yogjakarta at south Central Java in 2000. This outbreak and its ecology and epidemiology were described by Barcus and others^18^ and Figure 6

illustrates its progress.

**Figure 7.**
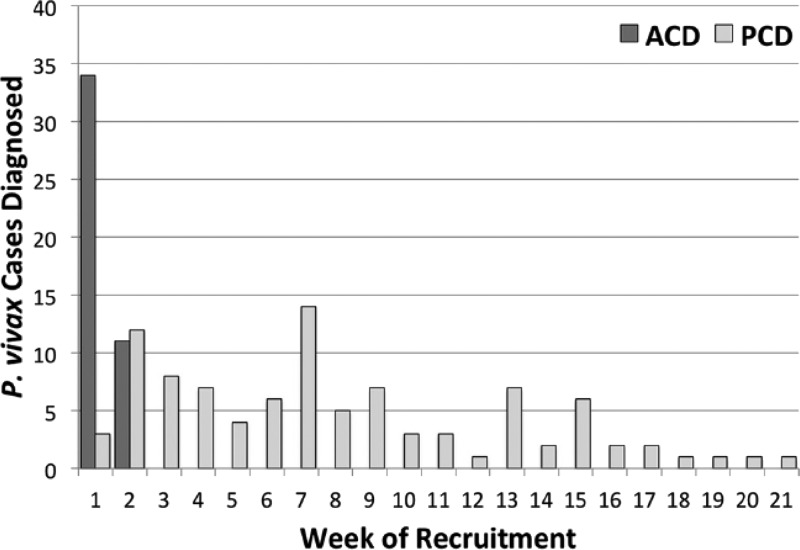
Case detection (active [ACD]; passive [PCD]) among 650 Indonesian soldiers during the first 5 months on Java after returning from 12 months of duty in northeastern Papua, Indonesia, reproduced from reference 7 with permission.

The threat of imported malaria is especially acute on Java, where 150 million of Indonesia's 250 million people reside. Javanese people migrate to the outer islands of Indonesia and frequently return home. Military populations represent one key piece of this much larger issue, as they routinely deploy to some of the most malarious areas of Indonesia and engage in relatively high-risk occupational exposure (e.g., sleeping in impermanent quarters and night patrols). This problem is especially acute with *P. vivax* because while those soldiers receive prompt and efficient diagnosis and treatment of acute malaria, they do not typically receive primaquine against hypnozoites for want of G6PDd screening and fear of causing harm. The screening and surveillance of one battalion of 650 men followed for 1 year after such a deployment netted only nine cases of falciparum malaria and 143 cases of vivax malaria.^7^ Moreover, most of those vivax cases did not appear at mass screening upon return to Java, but over the months that followed, that is, relapses. Figure 7

illustrates the timing of attack by relapse in those soldiers.

In another battalion of 532 men similarly deployed later and followed for clinical trial purposes, 18 soldiers were found to have *P. falciparum*, whereas 214 were diagnosed with acute *P. vivax*; again, most attacks occurred in the 4 months after deployment rather than immediately upon return.^19^ Chemoprophylaxis or presumptive radical cure of such may mitigate risk of chronic outbreaks at sites such as Java as Indonesia progresses in its elimination agenda.

Importation of malaria by travelers between the Outer Islands of Indonesia, especially those to the east, will continue to threaten success of the elimination of malaria from Java. In contrast, Bali also has very few isolated pockets of hypoendemic malaria (in the sparsely populated northwest of the island), but almost no problem with outbreaks. This may be linked to the relative prosperity of the Balinese and the relative infrequency of travel to other islands in search of economic opportunity compared with their Javanese neighbors.

Elyazar and others^10^ summarized malaria outbreaks in 2011 as follows: “Malaria outbreaks occur in Indonesia every year. For example, in 1998 and 1999 there were outbreaks in eight provinces, covering 10 districts with 19,483 cases and 66 deaths (case fatality rate, CFR 0.3%; Marwoto and Sekartuti, 2003). Between 2000 and 2005, there were outbreaks in 19 provinces, covering 65 districts/municipalities, with 58,152 malaria cases and 536 reported deaths (CFR 0.9%; Departemen Kesehatan, 2006c). In 2006, outbreaks occurred in eight provinces with 3705 cases and 30 reported deaths (CFR 0.8%; Departemen Kesehatan, 2007c). Later, between 2007 and 2008, outbreaks were reported in 11 provinces, covering 20 districts, with 1864 cases and 93 reported deaths (CFR 5%; Departemen Kesehatan, 2009a).” According to that author, *P. vivax* consistently dominates as the cause of these outbreaks (I. Elyazar, personal communication).

Malaria importation and outbreaks in receptive areas where malaria has been brought under control or eliminated is not only a serious public health problem, but also a direct threat to sustaining elimination where it has been achieved in Indonesia.

## Trends in Annual Parasite Incidence

The NMCP provided data on trends in annual parasite incidence (API) and annual proportions of reported cases represented by the two dominant species, *P. vivax* and *P. falciparum*. All five of the known plasmodia naturally infecting humans occur in Indonesia, but the other species are not reliably diagnosed and reported through routine surveillance. Nonetheless, reliable surveys or other research efforts provide assurance that these other species indeed represent a small minority of malaria cases in Indonesia (probably < 1%).

Figure 8

**Figure 8.**
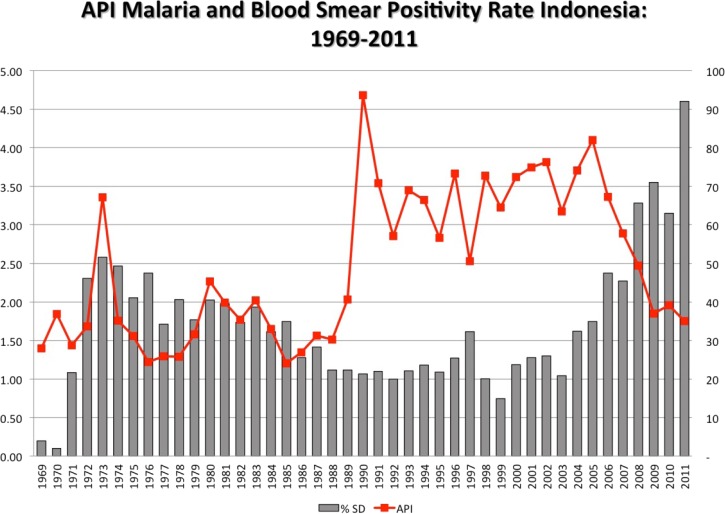
Annual parasite incidence (API = reported cases/1,000 total population) at the left axis, and the annual blood smear positivity rate (percent of smears examined positive for malaria, any species) at the right axis, from the Ministry of Health, Republic of Indonesia.

illustrates the API for all of Indonesia, along with the blood slide positivity rate. Further, the NMCP provided data regarding the proportion of reported cases (i.e., those represented by the API) represented by *P. vivax* and *P. falciparum*. Figure 9

**Figure 9.**
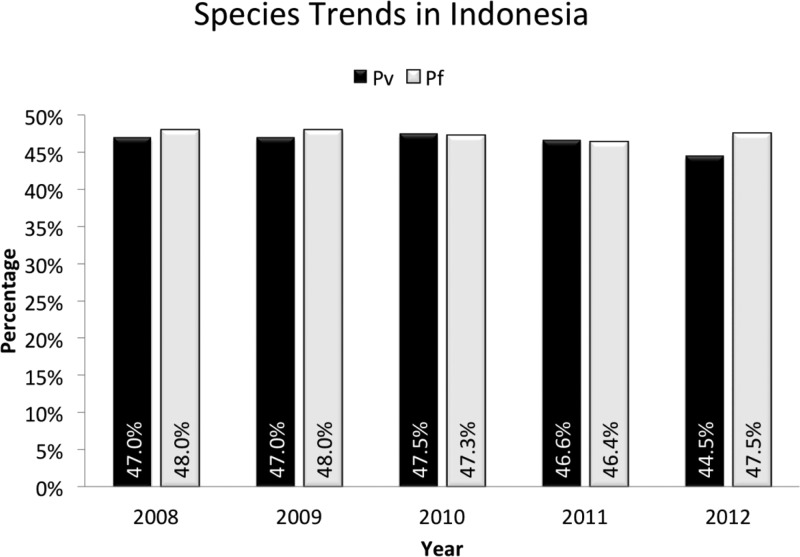
Species trend among cases reported to the Ministry of Health between 2008 and 2012, from the Ministry of Health, Republic of Indonesia.

illustrates those data. These data accord with those of the aggregated cross-sectional surveys shown in Table 1, that is, essentially equal risk of both of the dominant species in Indonesia as a whole and through the historical record of mass blood surveys (dating to 1899). Although substantial gains against malaria appear to have been made between 2005 and 2011 (with a 55% reduction in API), there is no evidence of increasing dominance of *P. vivax* as has been witnessed in many other settings in the wake of successful control programs.

## Relapse of *P. vivax*

**Figure 10.**
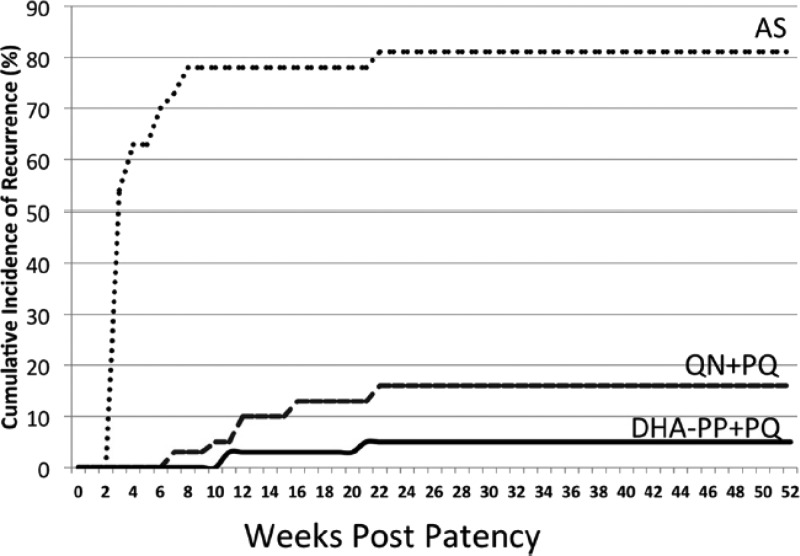
Timing of first relapse after treatment of an acute attack among soldiers returning from Papua to Java with either artesunate alone (AS), quinine, and 30 mg primaquine daily for 14 days (QN + PQ), or dihydroartemisinin–piperaquine and the same dose of primaquine (DHA-PP + PQ), reproduced from reference 7 with permission.

Relapse behaviors of *P. vivax* in Indonesia may be representative of what is generally believed to be the southeast Asian/west Pacific pattern represented by the single strain called Chesson.^20^ That strain was obtained from an American soldier probably infected in the area of Jayapura, Papua, during 1944, and later extensively studied in experimentally challenged human prisoner volunteers. The relapse behavior of that strain may be generalized as rapid and frequent, with most first relapses occurring at around day 21 postpatency of the primary parasitemia, 75% or more relapsing before day 28, and up to five relapses over 2 years being the rule.^5^ Studies in Thailand documented essentially similar relapse behaviors there in more recent times.^12^,^21^ Further, a recent trial in Indonesian soldiers diagnosed with *P. vivax* and permitted to relapse also showed Chesson-like relapse behaviors. Figure 10

illustrates the timing of first relapse with and without primaquine therapy.^7^ The median day of relapse was day 21, and 64% had relapsed by day 28. This study did not permit study of multiplicity of relapses, but the diagnosis of vivax malaria in these subjects was some weeks removed from their 12-month exposure to infection and very probably represented relapses rather than primary parasitemia. In other words, multiple relapses appear to be the rule with *P. vivax* from Papua (and Thailand).

One study of American soldiers infected in the Pacific theater of World War II provides a glimpse at multiplicity of relapses with endemic exposure in the region.^5^ Among the 659 soldiers evaluated, 213 suffered more than six relapses, and 105 of those more than 11. What all the available data indicate is that relapse is a very significant source of acute attacks of vivax malaria in Indonesia and that the failure to prevent it with primaquine therapy very probably contributes substantially to the disease burden imposed by *P. vivax*. Studies in neighboring Papua New Guinea lend substantial support for this hypothesis.^22^

## G6PD Deficiency in Indonesia

**Figure 11.**
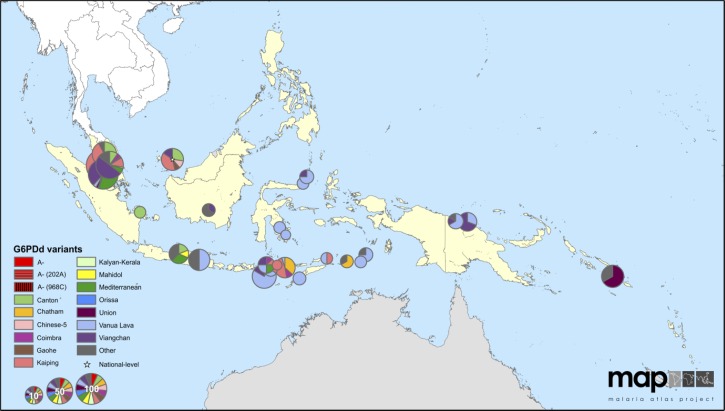
Frequencies and distributions of variants of glucose-6-phosphate dehydrogenase deficiency along the Indonesian archipelago, reproduced from reference 23, which was published under creative commons.

The very significant problem of relapse in Indonesia gives substantial weight to the directly linked problem of G6PD deficiency, that is, the phenomenon largely driving poor access to safe primaquine therapy. The great heterogeneity of G6PD deficiency in Asia^23^ is mirrored on the Indonesian archipelago, where several hundred distinct ethnolinguistic groups reside, each with its own pool of inherited G6PD deficiency types. It is impossible to generalize beyond “diverse” the variants of G6PD deficiency occurring in Indonesia, and the numbers of studies of such are limited. A map generated by Howes and others^23^ further illustrates what is known with respect to G6PD deficiency diversity in Indonesia (Figure 11

). Although the sampling on these data can only be described as sparse and inadequate to the area and human diversity inherent to Indonesia, one may draw some general conclusions regarding the variants represented. Vanua Lava variant seems to be especially common in the east and relatively dominant, whereas in the west, there seems to be greater diversity with Mediterranean, Viangchan, Union, and Kaiping variants all frequently represented.

**Figure 12.**
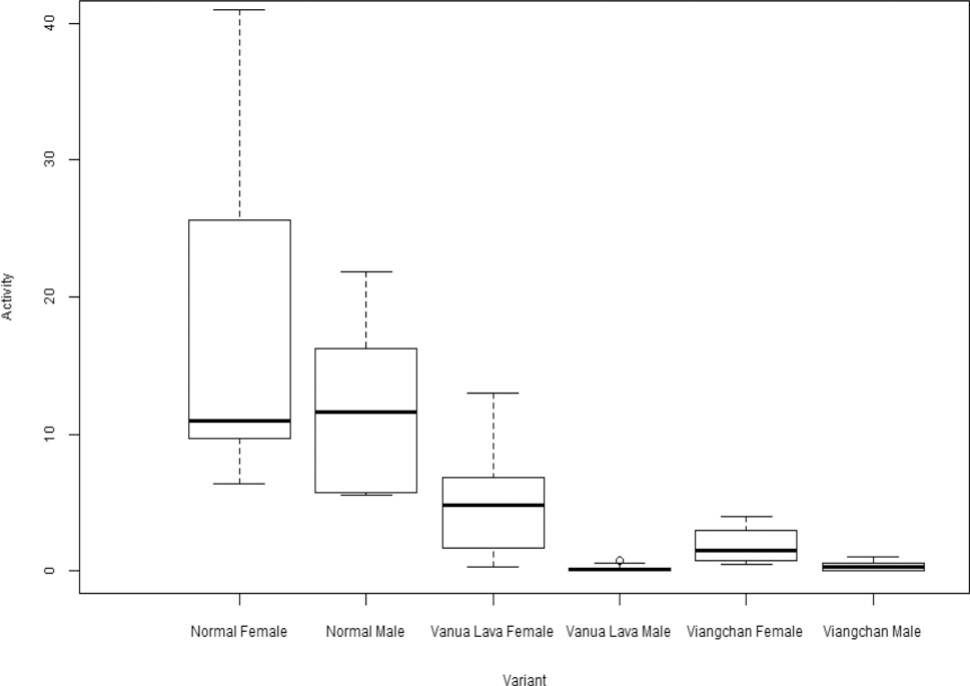
Quantitative glucose-6-phosphate dehydrogenase (G6PD) activities among normal and deficient male and female subjects represented by the three dominant variants in a survey of over 2,000 subjects resident in western Sumba Island in eastern Indonesia.^8^ The dark horizontal bars represents the mean G6PD activity (*y* axis, U/g Hb), the boxes show the interquartile range, and the dashed lines show the 95% confidence intervals. Courtesy of Ari Satyagraha, the Eijkman Institute for Molecular Biology, Jakarta.

Satyagraha and others^8^ evaluated G6PD activity phenotypes among residents of Sumba in eastern Indonesia and genotyped as Vanua Lava or Viangchan. Figure 12

summarizes those data. All these data provide reason for concern regarding primaquine therapy in Indonesia. Mediterranean variant is well known as being exquisitely sensitive to primaquine, and it occurs with some frequency in the west. In the east, both Vanua Lava and Viangchan (Figure 12) come with very low residual levels of enzyme activity (< 5%). In summary, primaquine sensitivity would appear to be relatively high and common in Indonesia. This question, however, requires a great deal more survey work before such generalizations may be considered reliable.

## Chemotherapy of *P. vivax* in Indonesia

### Therapeutic efficacy of standard therapies.

A number of clinical trials conducted at Timika, Papua, over the past 10 years provided a wealth of evidence of good efficacy of various ACTs against the chloroquine-resistant blood stages of *P. vivax* of that region.^24^–^27^ Those studies, along with others from many regions of Indonesia^28^,^29^ provide assurance of continuing good efficacy and safety with this class of therapeutics against acute vivax malaria.

There is much less assurance regarding the same for primaquine therapy. Only two trials of primaquine safety and efficacy have been reported from Indonesia.^7^,^19^ The therapeutic efficacy of primaquine against relapse when administered after DHA-PP was 98% and 95% in the published trials, but these both used 0.5 mg/kg daily dosing for 14 days, 2-fold dose recommended by the Ministry of Health. Concern regarding potential harm at the higher dose without G6PD screening explains that recommendation (see section Chemotherapeutic policy for vivax malaria).

### Chemotherapeutic policy for vivax malaria.

A high prevalence of chloroquine-resistant *P. vivax* in Indonesia (see section Drug-resistant *P. vivax* in Indonesia), prompted the Ministry of Health to recommend an ACT, DHA-PP, as first-line therapy against the acute attack. It also recommends concurrent primaquine therapy (0.25 mg/kg/day × 14 days).

The most recent treatment policy guidelines (2014) recommend antirelapse therapy without mention of G6PD deficiency screening. The guidelines instead offer a warning regarding signs and symptoms of acute hemolytic anemia in patients receiving primaquine (and other drugs), and recommend switching to a weekly dose of 0.75 mg/kg for 8–12 weeks should such signs appear with daily dosing. These policies largely align with those expressed in the new (2015) guidelines for treatment of malaria issued by the World Health Organization (WHO).^30^

### Drug-resistant *P. vivax* in Indonesia.

Resistance to chloroquine by *P. vivax* dominates most of the Indonesian archipelago. The problem seems to have originated in Papua sometime during the 1980s or perhaps earlier. Elyazar and others^10^ summarized surveys of such in Indonesia up to 2011 (Table 2 ). The report of resistance to chloroquine by *P. vivax* in Australian travelers to Papua New Guinea in 1989^31^ prompted studies in Indonesian Papua (Irian Jaya in that era) that affirmed a frequent and widespread problem in that far eastern province.^10^ During the 1990s, studies at Kalimantan, Sumatra, Java, and Lombok also revealed resistance to chloroquine, but at much lower frequencies.^10^ Isolates from Papua and Sumatra were later demonstrated as resistant to chloroquine in monkey models.^32^,^33^ In the decade that followed, some of these islands would also be described with high frequencies of chloroquine failure against *P. vivax*.^34^ The decision to abandon chloroquine for treatment of the acute attack was made in 2006.^35^

Resistance to primaquine is a highly complex and difficult question. In Indonesia, as elsewhere, it has yet to be demonstrated. This is not to say sensitivity to primaquine has been examined and proven as the rule. It is a remarkable fact that being the only therapeutic option against relapse of *P. vivax* since 1952, primaquine is only rarely evaluated for efficacy for its primary therapeutic indication. There is no systematic or standardized means of ascertaining a diagnosis of hypnozoites resistant to standardized doses of primaquine. A number of important confounding factors must be addressed, namely, adherence to the prolonged dosing, quality of drug, reinfection during the long period of risk of relapse, recrudescence by drug-resistant asexual blood stages, pharmacokinetic or pharmacodynamic interference with primaquine efficacy by untried partner blood schizontocides, and possibly polymorphisms in cytochrome-P450 2D6 isozyme.^36^ At present, we know only that a regimen of 0.5 mg/kg primaquine (daily for 14 days) administered with DHA-PP for radical cure exerts good efficacy against relapse of *P. vivax* acquired in eastern Indonesia.^7^,^19^ Efficacy of the lower standard dose, 0.25 mg/kg for radical cure is not currently known in Indonesia. Broader global surveys of recurrence trends postprimaquine tend to affirm poor efficacy of the lower dose regimen.^37^

### The primaquine–G6PD deficiency–*P. vivax* dilemma.

As in other *P. vivax*–endemic nations, Indonesia faces a therapeutic dilemma with primaquine due to its toxicity in patients with G6PD deficiency. Most providers face patients of unknown G6PD status and, absent such, must choose between potential harm caused by repeated relapses that follow the withholding of primaquine therapy, and potential harm in offering the treatment. The long misunderstanding of *P. vivax* as benign, and of treating relapse as futile where reinfection occurs, denied this dilemma the attention it required. The NMCP of Indonesia now actively explores options for coping with this dilemma.

Historically, the strategy has been to offer the less threatening daily regimen of 0.25 mg/kg with monitoring to mitigate the risks to G6PD-deficient patients. That approach suffers two key pitfalls: 1) probable poor efficacy and 2) poor ability to closely monitor patients where most acquire infection and seek treatment. Acknowledging that many or most variants of G6PD deficiency exhibit severely limited enzyme activity, suggests that strategy as both high risk and low benefit to the patients.

As WHO has very recently recommended,^38^ Indonesia now weighs implementing G6PD screening at the point of care. The hope is to safely provide primaquine to the majority in whom it offers tremendous health benefits with little or no risk of harm, while protecting the minority at risk of serious harm caused by that drug. Finance and roll out of G6PD screening is a challenge for Indonesia, but one offering very substantial gains with respect to national control and elimination goals. Acquiescence to the hypnozoite reservoir by an inappropriate status quo regarding primaquine therapy is considered no longer tenable.

## Vulnerable Populations

Pregnant women and infants cannot be offered primaquine, and some experts extend that prohibition to lactating women and children under 4 years of age. WHO recently clarified its recommendation concerning infants less than 6 months of age not to receive primaquine.^30^ Dellicor and others^39^ estimated the number of pregnancies in areas of endemic malaria transmission in Indonesia. In 2007, they estimated 6.4 million pregnancies (resulting in 3.8 million live births) occurred where any species of malaria was actively transmitted. These numbers take on special significance in light of the studies of Poespoprodjo and others^40^,^41^ at a hospital in Timika, Papua. They found vivax malaria to be an important cause of morbidity in infancy and in pregnant women. Further, McGready and others^42^ in Thailand (of over 17,000 pregnancies) concluded that febrile *P. falciparum* or *P. vivax* malaria during the first trimester of pregnancy elevated risk of spontaneous abortion by a factor of 4, regardless of the species. *Plasmodium vivax* threatens several million pregnant women, their fetuses, and their newborns every year in Indonesia. This fact, taken together with the lack of access to primaquine therapy against hypnozoites, should raise very serious concerns in the global health community. Indonesia, as many other nations, requires optimized and validated means of preventing relapse without primaquine in these highly vulnerable populations.

There are other vulnerable populations, as well, mostly those also lacking access to prompt diagnosis and safe and effective therapy for malaria for reasons of social, economic, or geographic isolation. Primary among these will be patients with G6PDd. The socially isolated may be communities of illegal miners or loggers. The economically isolated may be so impoverished as to be unable to afford even very inexpensive diagnosis and treatment services, or the transport necessary to access them. Likewise, many communities in Indonesia reside at isolated locations where access to care delivery is difficult, time consuming, and relatively expensive. These vulnerable and hard-to-reach populations may suffer chronic vivax malaria and the serious morbidity and mortality risks that come with such isolation. Recently, a young Ministry of Health physician died of vivax malaria at his isolated post in the highlands of Papua because weather prevented his evacuation to hospital by airplane (A. Surya, personal communication).

## Morbidity and Mortality Due to Vivax Malaria

There are no reliable estimates for morbidity or mortality burdens imposed by *P. vivax* in Indonesia. Reporting systems do not include degrees of illness, but presumably case reporting comes almost entirely from people seeking treatment of febrile illness at government-sponsored care delivery centers. As such, the API (already detailed) derived from these reporting systems provide some insight on disease burdens. The reported API for 2012 was 1.75/1,000; 256,592 reported cases of malaria. The WHO translated that number (through an estimate adjusting algorithm that takes reporting inefficiencies into account) to 5,453,703 cases for Indonesia in 2012.^43^ In 2013, Indonesia reported 170,848 cases confirmed as *P. falciparum* and 150,985 cases confirmed as *P. vivax*, reflecting the consistency of parity between the two dominant species with respect to both prevalence surveys over the decades (Table 1), and API over recent years (Figure 10). The burden of morbidity imposed by *P. vivax* in Indonesia may be reasonably presumed to approximate that of *P. falciparum*. In 2011, the Malaria Atlas Project estimated the burden of clinical cases of *P. falciparum* malaria for Indonesia in 2010 at 12.3 million (95% confidence interval [CI] = 6–21).^44^ In contrast, the WHO estimate for all species of malaria in Indonesia during that year was just under 2 million cases. This sort of discordance is also seen in the global estimates of the burden of *P. vivax* attacks, that is, about 18 million cases estimated by WHO^38^ versus 70–390 million by several other groups.^45^ The great disparity in estimating morbidity among methodologies reveals the extraordinary difficulty of doing so. Work is in progress to estimate the burden of clinical disease imposed by *P. vivax* globally using the Bayesian statistical modeling of prevalent parasitemia.^45^

Even greater uncertainty haunts estimates of mortality due to malaria in Indonesia and elsewhere. In 2011, the NMCP recorded 388 deaths due to malaria and the WHO put the estimated mortality at 8,631 for that year.^43^ Indonesia's Central Bureau of Statistics conducted nationwide health surveys in 1995 and 2001 and estimated that 30,000 to 38,000 Indonesians died of malaria in each of those years.^10^ Similarly, although WHO reported approximately 15,000 deaths due to malaria in India in 2010, a systematic survey of mortality by verbal autopsy generated an estimated 210,000 deaths annually due to malaria in that nation.^46^

Although absolute numbers of fatalities due to *P. vivax* may evade credible estimation for now, several hospital-based studies carried out in Indonesia since 2005 leave no doubt that death often occurs in association with a diagnosis of *P. vivax* malaria.^2^ The data reported by Nurleila and others^47^ from a hospital at Sumba in eastern Indonesia may be typical. In that retrospective study, patients admitted with a diagnosis of *P. falciparum* were 2.9 times more likely to be classified as seriously ill compared with *P. vivax*. However, once classified as seriously ill, the odds ratio (OR) of not surviving was equal between the two species (OR = 1.3; 95% CI = 0.7–2.5). In the very large prospective study at a hospital at Timika, Papua, Tjitra and others^48^ observed equal rates of severe malaria between the species (0.8% versus 0.7% of admissions for *P. vivax* and *P. falciparum*), and also found the odds of death with a diagnosis of *P. vivax* indistinguishable from a diagnosis of *P. falciparum* (adjusted OR = 1.13, *P* = 0.51). These trends raise the possibility of *P. vivax* contributing a very substantial share of the mortality imposed by malaria in Indonesia.

Note that 64 deaths attributed to malaria occurred at the hospital at Sumba during 2008 and 2009.^47^ This number was considered ordinary by the hospital staff, and yet for the entire province of Nusa Tenggara Timor (Sumbawa, Flores, Sumba, Alor, Savu, and other areas), only 48 deaths were reported to the NMCP during the 3-year period, 2010–2012 (Table 3 ). Even patients admitted to hospital with a diagnosis of malaria and not surviving are somehow lost to mortality reporting systems. Improved surveillance of morbidity and mortality with a primary diagnosis of malaria at both treatment centers and hospitals are needed.

## Challenges to Elimination

*Plasmodium vivax* seriously challenges health in Indonesia. The long misunderstanding of this species as benign and not threatening drove its neglect by the communities of science, medicine, and public health. The tools needed to combat this threat were not developed, especially a means to safely and effectively attack the tenacious and dangerous hypnozoite reservoir residing in endemic communities. Overcoming the serious problem of primaquine toxicity in G6PD-deficient patients, and thus greatly improving access to this crucial therapy is a top priority in Indonesia.

The NMCP faces challenges in meeting Indonesia's aspirations to eliminate malaria transmission by 2030. A highly mobile population and natural receptivity to malaria transmission across all of Indonesia's islands will demand decades of diligent surveillance and provision of expert diagnosis and treatment services. The dormant and silent hypnozoite is especially threatening in this respect, striking suddenly and unexpectedly months or years after exposure. Further, *P. vivax* circulates its infectious gametocytes even before patients become ill. Health services against malaria must be maintained even after transmission ceases.

Patients unable to receive primaquine pose another important challenge. Pregnant women or lactating women and their infants, who are especially vulnerable to *P. vivax*, must be provided alternatives to primaquine to prevent repeated clinical attacks. The same will be true of the many patients diagnosed as G6PD deficient. Chemoprophylactic or presumptive therapeutic regimens will need to be developed, optimized, validated, and implemented for these patients.

The many people living in geographic isolation across Indonesia also pose a special challenge to the NMCP. They need knowledge, tools of personal protection, diagnostics, and safe and effective therapies put into their hands. Eliminating malaria will require reaching well beyond where health-care providers live and work. Delivery of innovative implements against malaria to those isolated from health services would greatly advance the elimination agenda in Indonesia.

## Conclusions

*Plasmodium vivax* occurs all across Indonesia as a codominant species with *P. falciparum*. Both impose substantial burdens of morbidity and mortality across an enormously diverse landscape of anopheline vectors, habitats, and human genetic diversity. Indonesian *P. vivax* is widely resistant to chloroquine, tolerates lower doses of primaquine therapy, exhibits the aggressive relapse behavior of Chesson-like strains, and may have selected for some of the most severely deficient G6PD variants known. Indonesia's malaria research community already works hand in hand with the NMCP to address these challenges, and progress has and will continue to be made against this very stubborn and difficult parasite.

## Figures and Tables

**Table 1:** Results of mass blood surveys recorded in Indonesia between 1900 and 2009

Islands	Year of sample	No. of sites	No. examined	No. of *Plasmodium falciparum* (%)	No. of *Plasmodium vivax* (%)	No. of *Plasmodium malariae* (%)	No. of *Plasmodium ovale* (%)
Sumatra	1919–2009	676	239,109	8,487 (3.5)	7,057 (2.9)	494 (0.2)	–
Java/Bali	1900–2006	114	105,734	3,387 (3.2)	2,773 (2.6)	221 (0.2)	–
Kalimantan	1975–2005	17	7,367	398 (5.4)	248 (3.4)	21 (0.3)	–
Sulawesi	1972–2006	55	11,530	482 (4.2)	316 (2.7)	8 (0.1)	–
Maluku	1997–2009	201	121,526	5,311 (4.4)	13,198 (10.9)	3 (0.002)	–
Lesser Sundas	1975–2009	609	383,950	23,502 (6.1)	19,401 (5.1)	157 (0.04)	11 (0.003)
Papua	1929–2009	694	193,043	19,848 (10.3)	9343 (4.8)	1,395 (0.7)	40 (0.02)
Indonesia	1900–2009	2366	1,062,259	61,415 (5.8)	52,336 (49)	2,299 (0.2)	51 (0.005)

Reproduced from reference 10 with permission.

**Table 2:** Summary of all in vivo tests for resistance to chloroquine in *Plasmodium vivax* in Indonesia between 1974 and 2010

Islands	Year of sample	In vivo test			
		No. of sites	No. examined	No. of resistance	Resistance (%)
Sumatra	1974–2010	5	67	20	30
Java/Bali	1996–2002	2	91	11	12
Kalimantan	1998	1	27	12	44
Sulawesi	1998	1	11	1	9
Maluku	–	–	–	–	–
Lesser Sundas	1975–2009	6	87	37	43
Papua	1991–2008	12	404	250	62
Total	1974–2010	27	687	331	48

Reproduced from reference 10 with permission.

**Table 3:** Summary of reported deaths due to malaria by province in Indonesia during 2010–2012, from the Ministry of Health, Republic of Indonesia

Province	Deaths caused by malaria		
	2010	2011	2012
Aceh	6	5	1
North Sumatra	0	71	65
West Sumatra	1	0	2
Riau	2	7	2
Riau Islands	3	7	0
Jambi	38	16	5
Bengkulu	0	4	14
South Sumatra	2	0	0
Bangka Belitung Islands	39	49	24
Lampung	11	1	5
West Nusa Tenggara	0	1	11
East Nusa Tenggara	30	11	7
Maluku	0	0	10
North Maluku	16	27	6
West Papua	61	17	3
Papua	69	99	51
North Sulawesi	3	0	0
West Sulawesi	5	1	1
Sulawesi Tenggara	19	21	8
Central Sulawesi	0	5	1
South Sulawesi	0	0	0
Gorontalo	6	2	7
Central Kalimantan	25	0	10
West Kalimantan	0	12	5
East Kalimantan	0	7	5
South Kalimantan	96	21	9
West Java	0	0	0
Central Java	0	1	0
East Java	0	3	0
Yogyakarta	0	0	0
Banten	0	0	0
Bali	0	0	0
Jakarta	0	0	0
Total	432	388	252
